# Supplementary material for: Massively parallel profiling and predictive modeling of the outcomes of CRISPR/Cas9-mediated double-strand break repair
Source: Nucleic Acids Res. 2019 Jun 5;47(15):7989–8003. doi: 10.1093/nar/gkz487 (PMC6735782; doi:10.1093/nar/gkz487)
Supplement: gkz487_Supplemental_File [file gkz487_supplemental_file.pdf]

SUPPLEMENTARY FIGURES

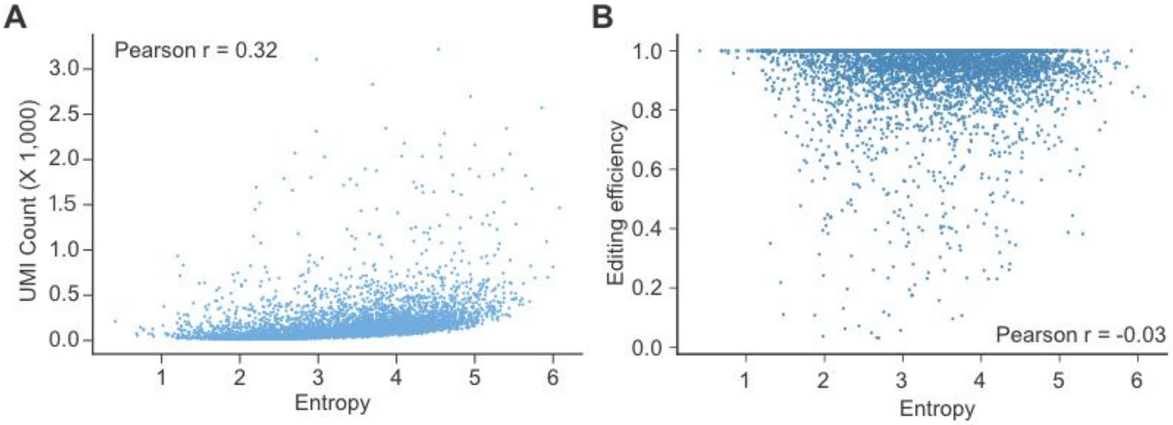

Figure S1. Correlation between entropy vs. UMI count or editing efficiency. (A) Estimates of target-specific entropy (x-axis) are only modestly correlated with UMI counts (y-axis). Pearson r = 0.32. **(B)** Estimates of target-specific entropy (x-axis) are not correlated with editing efficiency (y-axis). Pearson r = -0.03.

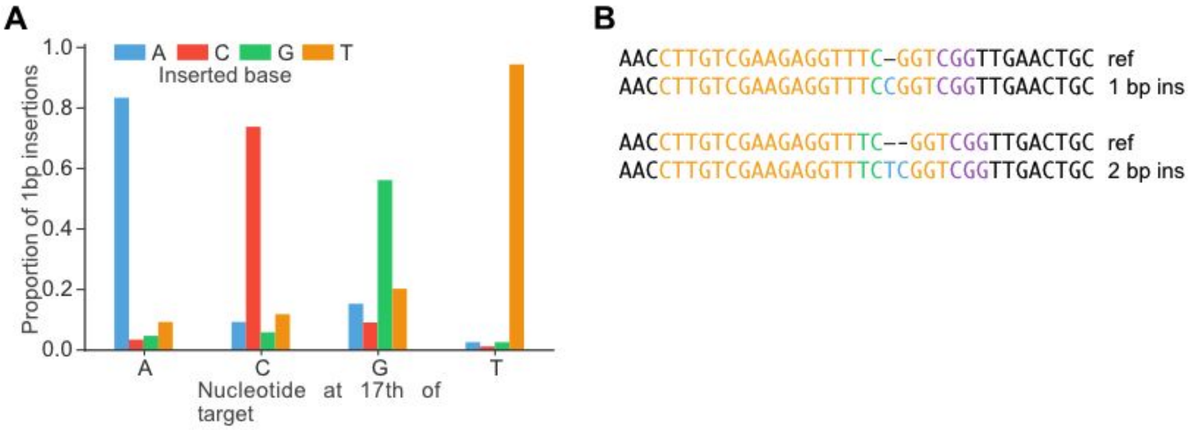

Figure S2. 1-2 bp insertion events are templated by the nucleotides upstream of the cleavage site. (A) Most 1 bp insertions were predicted, and presumably templated, by the identity of the 17th nucleotide of the target sequence. **(B)** Example of insertions templated by the 17th (top) or 16th and 17th (bottom) position. Template nucleotides are shown in green and inserted nucleotides are shown in blue.

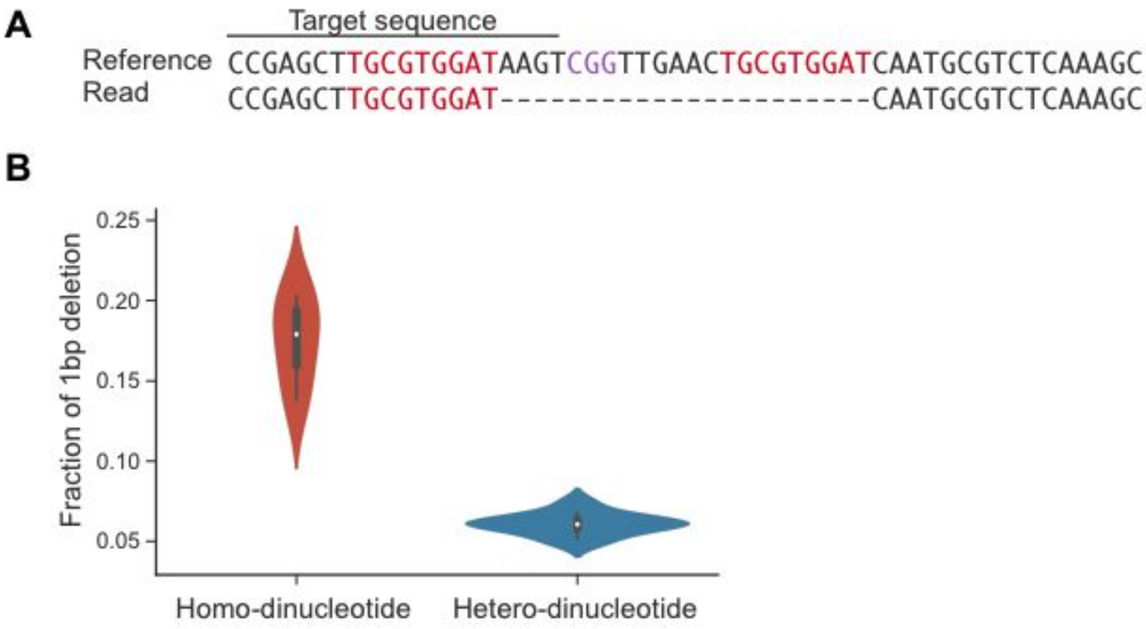

Figure S3. Examples of microhomology usage. (A) An observed example of a long MH tract mediating a deletion event. PAM and microhomology are shown in purple and red, respectively. This particular outcome, involving a 9 bp MH tract, represented 9% of indel events associated with this target. **(B)** Targets with identical nucleotides (*i.e.* 'homo-dinucleotide') spanning the cleavage site exhibit a much higher proportion of 1 bp deletions than targets with non-identical nucleotides (*i.e.* 'hetero-dinucleotide') spanning the cleavage site, suggesting that 1 bp microhomology may help mediate 1 bp deletion events.

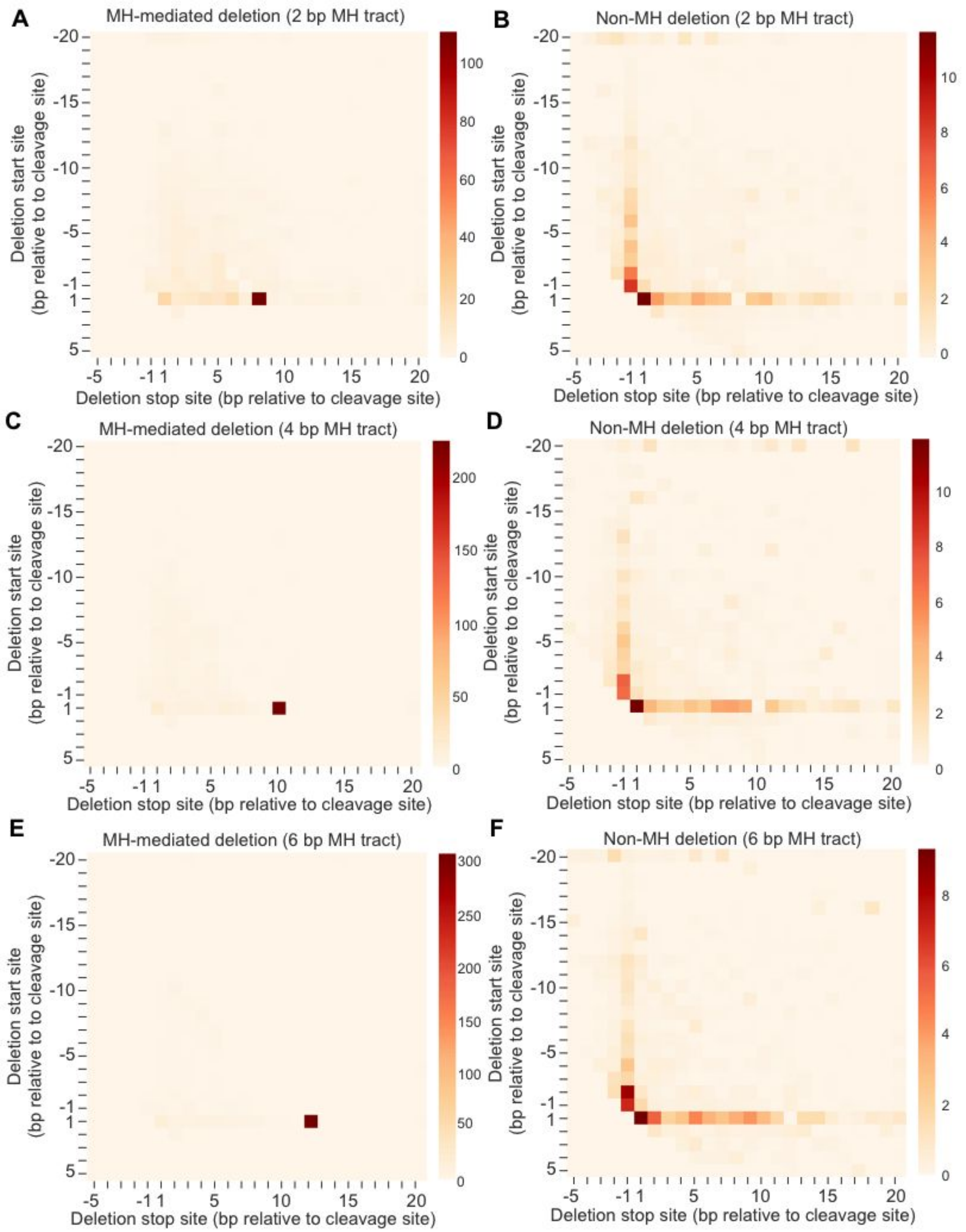

Figure S4. Heatmap of deletions with microhomology design. **(A,C,E)** Heatmap of showing frequency of start/stop sites of MH-mediated deletions with 2 bp, 4 bp, 6 bp programmed microhomology, respectively. **(B,D,F)** Heatmap of showing frequency of start/stop sites of non-MH deletions with 2 bp, 4 bp, 6 bp programmed microhomology, respectively.

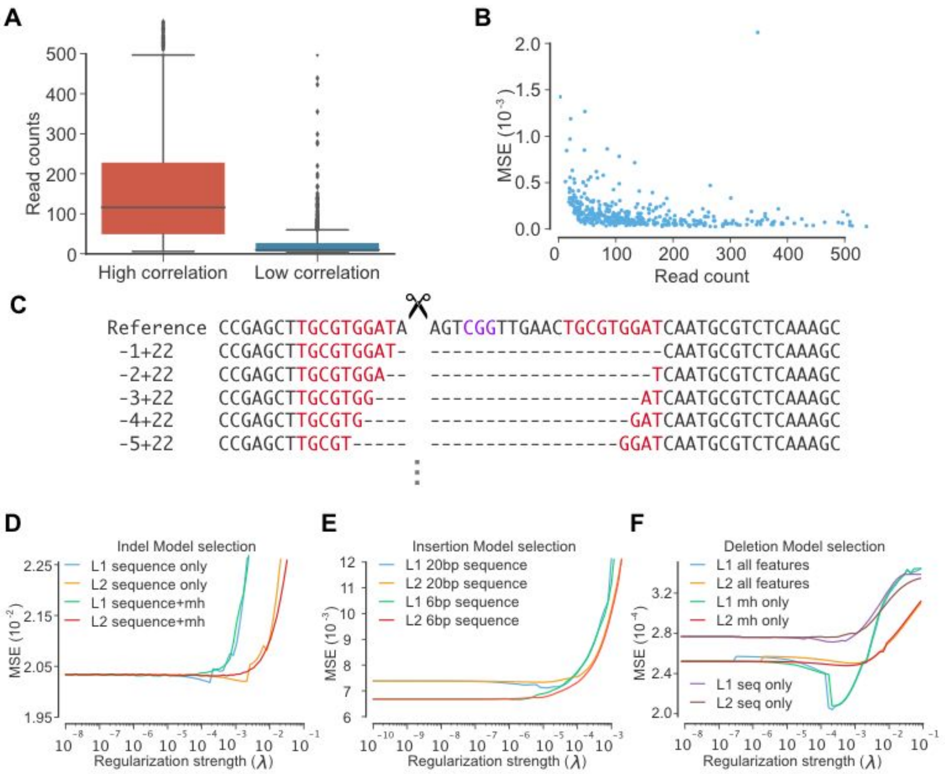

Figure S5. Machine learning model selection and performance. (A) Read counts for targets exhibiting high (r > 0.75) vs. low (r < 0.75) correlation between replicate experiments. The median read count for the two groups are 117 and 23, respectively. Targets with low correlation between replicates (r < 0.75) were excluded from model training/validation/testing. **(B)** Poorly predicted targets (high MSE) largely corresponded to those that were poorly sampled. **(C)** Example of redundant deletion classes. We define deletion classes using the deletion start site and deletion length (*e.g.* -1 + 22 where -1 is the location relative to the cleavage site and 22 is the deletion length). For any given sequence, there may be several deletion classes that represent identical outcomes due to microhomology (red). We collapsed the probability of these classes in prediction. PAM is colored in purple. **(D-F)** Model selection for indel ratio prediction, insertion prediction and deletion prediction. Hyperparameter search involved separate scans over regularization strengths for L1-regularization and L2-regularization individually with a range of 10^{-10} to 10^{-1}. MSE on the validation set is plotted and was used to pick the best performing model.

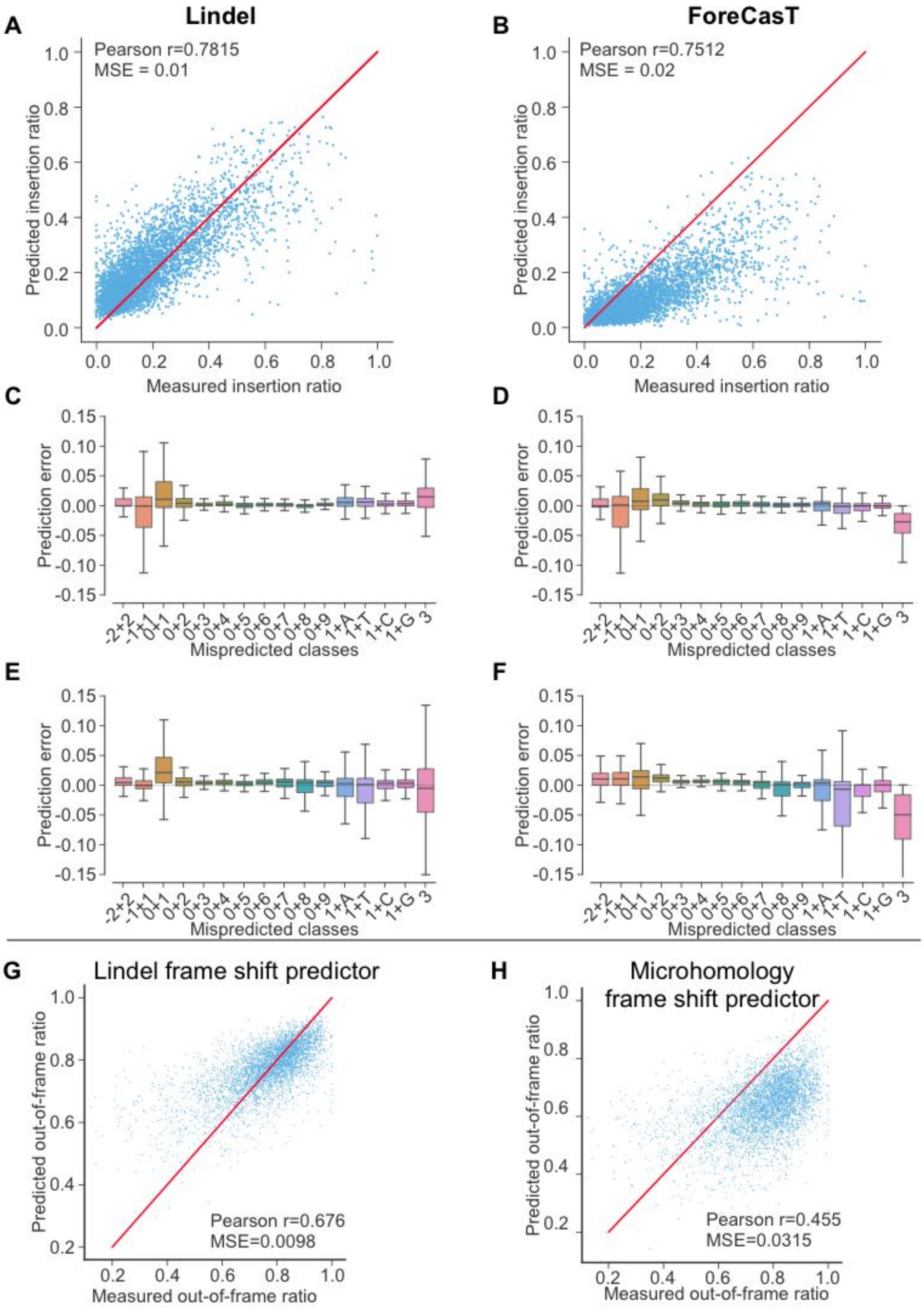
A
Lindel
Pearson r=0.7815
MSE = 0.01
Predicted insertion ratio
Measured insertion ratio
B
ForeCasT
Pearson r=0.7512
MSE = 0.02
Predicted insertion ratio
Measured insertion ratio
C
Prediction error
Mispredicted classes
D
Prediction error
Mispredicted classes
E
Prediction error
Mispredicted classes
F
Prediction error
Mispredicted classes
-2+2 -1+1 0+1 0+2 0+3 0+4 0+5 0+6 0+7 0+8 0+9 1+A 1+T 1+C 1+G 3
G
Lindel frame shift predictor
Pearson r=0.676
MSE=0.0098
Predicted out-of-frame ratio
Measured out-of-frame ratio
H
Microhomology
frame shift predictor
Pearson r=0.455
MSE=0.0315
Predicted out-of-frame ratio
Measured out-of-frame ratio

Figure S6. Performance of Lindel on ForeCast test I actuset. (A, B) Lindel (A) compared favorably to ForeCasT (B) in predicting the overall indel ratio for each of the 4,298 targets. **(C-F)**. Mispredicted classes in Lindel and ForeCast on both test set (C, E Lindel on ForeCast test set and our test set; D, F ForeCasT on their test set and our test set). Each boxplot summarized the error of certain classes. The box represents 25th percentile, 50th percentile and 75th percentile and whiskers represents 1.5x of the inter-quartile range (IQR). Small deletions around the cleavage site and 1 bp are difficult to predict accurately. **(G, H).** Lindel (G) compared favorably to Microhomology Predictor (34) (H) in predicting the ratio of frameshifting mutations for each of the 4,298 targets.

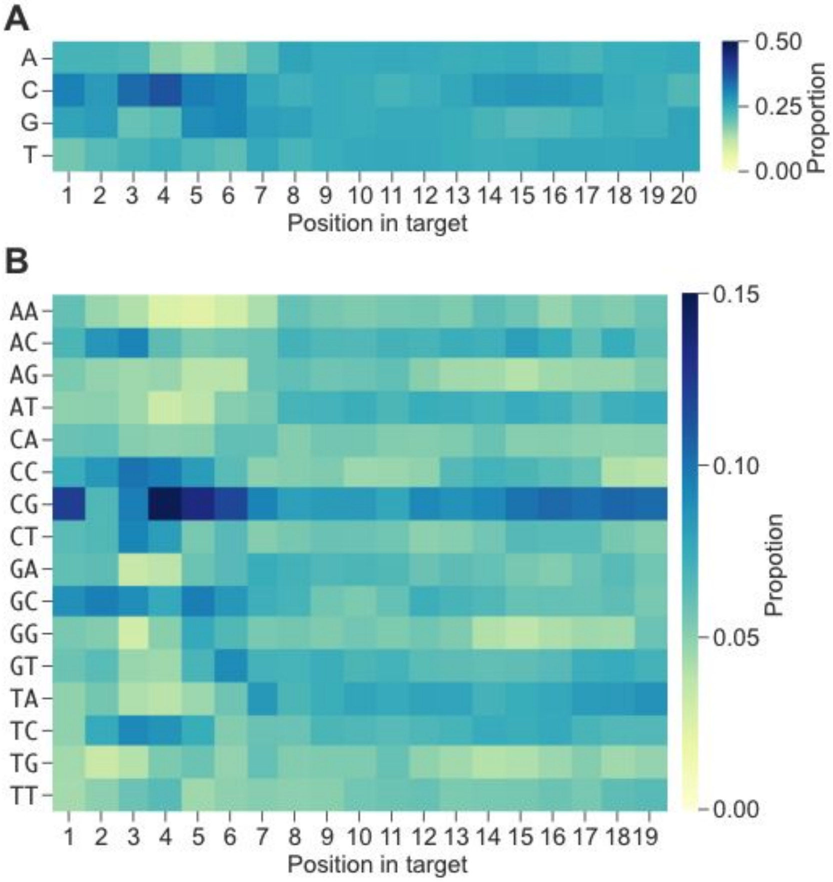

Figure S7. Sequence content of synthetic sgRNA-target library. **(A, B).** Heatmaps of mononucleotide (A) and dinucleotide (B) balance within the final subsampled library of 6,872 well-represented CRISPR/Cas9 targets on which most analyses were performed. Each column sums to 1. Although initially designed sequences were balanced in mono/dinucleotide content, the overrepresentation of CG dinucleotides was likely introduced by how we screened these initial designs to remove on-target or off-target matches against the human genome (*i.e.* thereby subtly selecting in favor of designs containing CG dinucleotides, which are underrepresented in the human genome).

Table S1. Comparison of the design and results of different profiling data

	Total targets tested	Endogenous/genomic targets	Synthetic targets	Cell line(s)	Indels predicted
This study	6,872	0	6,872	HEK293T	536 classes of deletions (~420 unique)<br>21 classes of insertions
Shen et al. (inDelphi)	1,872	0	1,872	HEK293,K562, HCT116, mESCs and U2OS	~90 classes of MH-mediated deletion<br>59 classes of Non-MH deletion<br>4 classes of 1bp insertion
Allen et al. (ForeCasT)	41,630	6,654	27,906 unique + others	K562, RPE-1, iPSC, CHO, HAP1 and mESCs	~420 classes of deletions (slightly varied by sequence)<br>20 classes of insertions
Chakrabarti et al.	1,492	1,492	0	HepG2	Not available

Table S2. Primers. Heatmap of deletions with microhomology design

**Name**	**Sequence**	**Usage**
P1	5' AAGCTTGGCGTAACTAGATCTTGAGACAAA 3'	Backbone cloning
P2	5' ATTTACAACCGTCTCCGGTGTTTCG 3'	Backbone cloning
P3	5' TTGAGACATTGGTGGACGCGTCGTCTCAAAGCTTGGCGTAACTAGATC 3'	Backbone cloning
P4	5' ACGCGTCCACCAATGTCTCAAATTTACAACCGTCTCCGGTGTTTCG 3'	Backbone cloning
P5	5' GAGCAGCTCGTCTCTCACC 3	Oligo amp
P6	5' GCAAGCTTTGAGACGCATTG 3'	Oligo amp
P7	5' GCGTCAGATGTGTATAAGAGACAGNNNNNNNNNNNNNNNGGCTTTATATATCTTGTGGAAAGGACGAAACACCG 3'	UMI annealing
P8	5' GCGTCAGATGTGTATAAGAGACAG 3'	Genomic DNA amp up
P9	5' TTCAGACGTGTGCTCTTCCGATCTGTCTCAAGATCTAGTTACGCCAAGCTTTGAGACGC 3'	Genomic DNA amp up
P10	5' TTCAGACGTGTGCTCTTCCGATCTGTGGATGAATACTGCCATTTGTCTC 3'	Genomic DNA amp up
P11	5' AATGATACGGCGACCACCGAGATCTACACNNNNNNNNNNTCGTCGGCAGCGTCAGATGTGTATAAGAGACAG 3'	Sequencing adaptor Nextseq I5
P12	5' CAAGCAGAAGACGGCATACGAGATNNNNNNNNNNGTGACTGGAGTTCAGACGTGTGCTCTTCCGATCT 3'	Sequencing adaptor Trueseq I7

Table S3. Statistics of sequencing runs

Sequence library	Reads	UMIs	UMI pass filter	Reads pass filter	UMI with designed sequence	Template switch	Other mutations	Final UMI count
Replicate 1	42,796,114	8,393,602	1,327,837	27,896,784 (65%)	1,005,606	27.15%	37.28%	357,671 (35.57%)
Replicate 2	60,898,224	12,094,298	1,783,500	39,107,335 (64%)	1,354,159	26.98%	37.27%	484,056 (35.75%)
Replicate 3	44,075,664	9,794,807	1,294,042	24,321,581 (55%)	982,804	27.92%	36.56%	349,066 (35.52%)
Total of Replicates 1-3	147,770,002							1,190,615
MH design	31,239,645	6,266,832	659,220	20,810,016 (67%)	445,440	8.05%	36.04%	249,039 (55.91%)
